# Effect of elevated beverage temperatures on the physical and mechanical properties of invisalign clear aligners: an in-vitro simulation study

**DOI:** 10.1007/s44445-025-00026-x

**Published:** 2025-06-11

**Authors:** Athar Alweneen, Nasser Alqahtani

**Affiliations:** Department of Pediatric Dentistry and Orthodontics, College of Dentistry, King Saud University, 11545 Riyadh, Saudi Arabia

**Keywords:** Clear aligners, Elastic modulus, Hardness, Invisalign, Temperature, Thermocycling

## Abstract

Although guidelines recommend removing aligners before eating, many patients wear them while consuming food or beverages. Understanding the response of a material to high temperatures is crucial for predicting treatment outcomes. This study aimed to assess the effects of elevated beverage temperatures on the physical and mechanical properties of Invisalign clear aligners. Sixty Invisalign aligner specimens were thermocycled and divided into four groups. The specimens in the first and second groups were immersed in coffee and tea at 57 °C, respectively, whereas the third and fourth groups consisted of raw and thermocycled specimens, respectively. Each specimen from group 1 and 2 was immersed in each solution and subsequently in artificial saliva to simulate an intermittent drinking process, which was repeated 200 times, with each immersion lasting 2 s. The elastic moduli and hardnesses of the materials were measured and compared with those of the raw and thermocycled specimens. Statistically significant differences were observed in mean hardness and elastic modulus values (*p* < 0.0001 and *p* = 0.025, respectively). The mean hardness of the raw specimens was significantly higher than those of the other three groups (*p* < 0.0001 each), and the elastic modulus was lower than that of the coffee group (*p* = 0.018) but not statistically different from those of the thermocycling and tea groups (*p* = 0.413 and *p* = 0.309, respectively). Thermocycling and exposure to beverages at 57 °C significantly decreased the hardness of Invisalign clear aligners. The coffee-exposed group exhibited an increased elastic modulus, indicating greater rigidity. Investigating the effects of increased temperature on thermoplastic materials is crucial to ensuring the durability and safety of orthodontic treatment, which directly impacts patient care.

## Introduction

An increase in adult orthodontic patients has resulted in a greater demand for aesthetically pleasing and comfortable alternatives to traditional fixed orthodontic appliances (Alhafi et al. 2024). Newly developed transparent thermoplastic removable appliances have gained widespread acceptance in clinical orthodontics (Iijima et al. 2015). These devices work by gradually shifting the target tooth as the patient progresses through a series of trays (Barone et al. 2017). The major advantages of clear aligner treatment are improved aesthetics, higher patient acceptance, and an overall improvement in quality of life (Fujiyama et al. 2014). However, the efficiency of clear aligners in complex cases remains unclear, and understanding their full interactions with the oral environment is important (Baneshi et al. 2025).

Various materials, including polyethylene terephthalate glycol (PeT-G), polypropylene (PP), polycarbonate (PC), thermoplastic polyurethanes (TPU), and ethylene–vinyl acetate (EVA), are used to manufacture aligners (Macrì et al. 2022) High-grade thermoplastic polyurethane is typically used to create transparent aligners through the thermoforming process on digital models (Thavarajah and Thennukonda 2015). Polymers are composed of long chains of organic units connected by urethane bonds (Wible et al. 2019). The use of synthetic polymers has several drawbacks, including the potential to cause undesirable biological reactions in live tissues when the remaining monomers seep into saliva in the oral cavity (Alhendi et al. 2022).

The molecular structure and branching patterns of polymers significantly influence their thermal characteristics. The polymers used for orthodontic aligners are semi-crystalline, consisting of sections of highly organized crystalline segments interspersed with amorphous regions. The proportions of the two regions affect both the mechanical and the thermal properties of the material, with a greater proportion of crystalline regions resulting in a more rigid material with an elevated glass transition (Tg) temperature (Balani et al. 2014). The Tg at which the rigid state of the aligner material transforms into a rubbery state is around 80 °C, which is much higher than the accepted oral temperature (Bichu et al. 2023). However, a temperature change in the oral cavity above 57 °C after consuming hot beverages affects the mechanical properties of thermoplastic materials (Iijima et al. 2015).

Fluctuations in oral temperature may affect the mechanical properties of thermoplastic materials (Kwon et al. 2008). The mechanical properties of thermoplastic materials have been shown to be altered by water absorption, thermoforming, and temperature fluctuations, all of which lead to a decrease in crack resistance. After consuming a hot beverage, the oral environment temperature may reach 57 °C, and it may take several minutes to return to normal. This increase in temperature can affect the mechanical characteristics of thermoplastics (Airoldi et al. 1997). Therefore, it is important to investigate how increased temperature and moisture affect thermoplastic material characteristics (Iijima et al. 2015).

Hardness is the resistance to plastic deformation, measured in terms of force per unit area (Sakaguch et al. 2019). The hardening of thermoplastic materials during clinical use can cause pain and alter their force delivery capabilities (Schuster et al. 2004). The elastic modulus (Young's modulus) is a critical mechanical property that measures a material's stiffness and its ability to withstand elastic deformation under stress (Cowley et al. 2012). Materials with low elastic modulus are less brittle than materials with high elastic modulus, and they exert lower elastic forces (Siotou et al. 2025). In addition, research has found that the hardness and elastic modulus of thermoplastic materials undergo different changes when subjected to thermocycling tests, and further tests have been conducted to evaluate their mechanical properties (Iijima et al. 2015).

The effect of hot beverages on the mechanical properties of clear Invisalign aligners has not yet been explored. This raises the question of whether the elastic modulus and hardness of the aligner material vary upon exposure to high beverage temperatures. Therefore, this study aimed to assess the effects of elevated beverage temperatures on the hardness and elastic modulus of Invisalign clear aligners. We aimed to determine whether elevated beverage temperatures have any significantly effect on the mechanical properties of clear Invisalign aligners.

## Materials and methods

Based on a statistical power of 0.85 and an alpha level of 0.05, a total of 60 specimens were determined to be necessary to detect an effect size of 0.71. Specimens with a 6-mm diameter were cut from Invisalign aligners in the central incisor area to obtain flat sheets using an Invisalign puncher (Align Technology, Santa Clara, CA, USA).

### Thermocycling

**Fig. 1:**
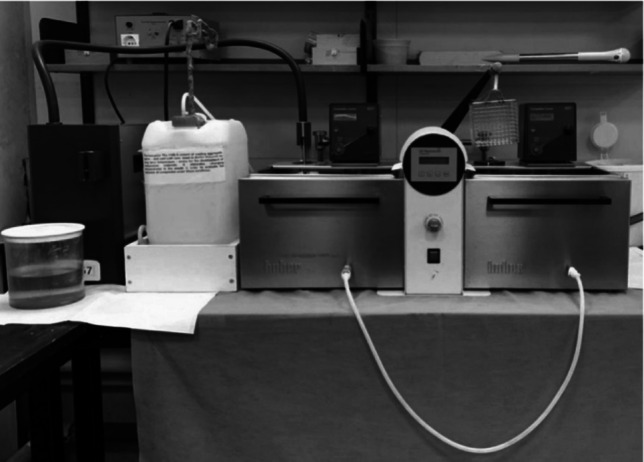
Thermocycler 1100 (SD-Mechatronik, Westerham, Germany)

All specimens were thermocycled in distilled water using a Thermocycler 1100 (SD-Mechatronik, Westerham, Germany) at a temperature ranging between 5 °C and 55 °C for 500 cycles, with each cycle lasting 15 s to mimic the intraoral environment. This process was simulated approximately to 14 days of clinical use (Fig. 1).

### Preparation of artificial saliva

Artificial saliva was prepared by combining 100 mL of KH_2_PO_4_, Na_2_HPO_4_, HKCO_3_, NaCl, and MgCl_2_ + 6H_2_O. Subsequently, 8 mL of citric acid was added, followed by 10 mL of CaCl_2_. The solution was then diluted with distilled water until the total volume reached 1 L. The pH range of the final solution, determined using a pH meter, was 6.7–7.3. The compositions and levels of the components are listed in Table 1 (Alrahlah et al. 2018).

**Table 1:** Artificial saliva ingredients

Ingredient	Concentration (G/100 mL)
KH_2_PO_4_	0.3402
Na_2_HPO_4_	0.4450
HKCO_3_	1.5017
NaCl	0.5844
MgCl_2_ + 6H_2_O	0.0305
Citric Acid	0.5224
CaCl_2_	0.2205

### Testing

**Fig. 2:**
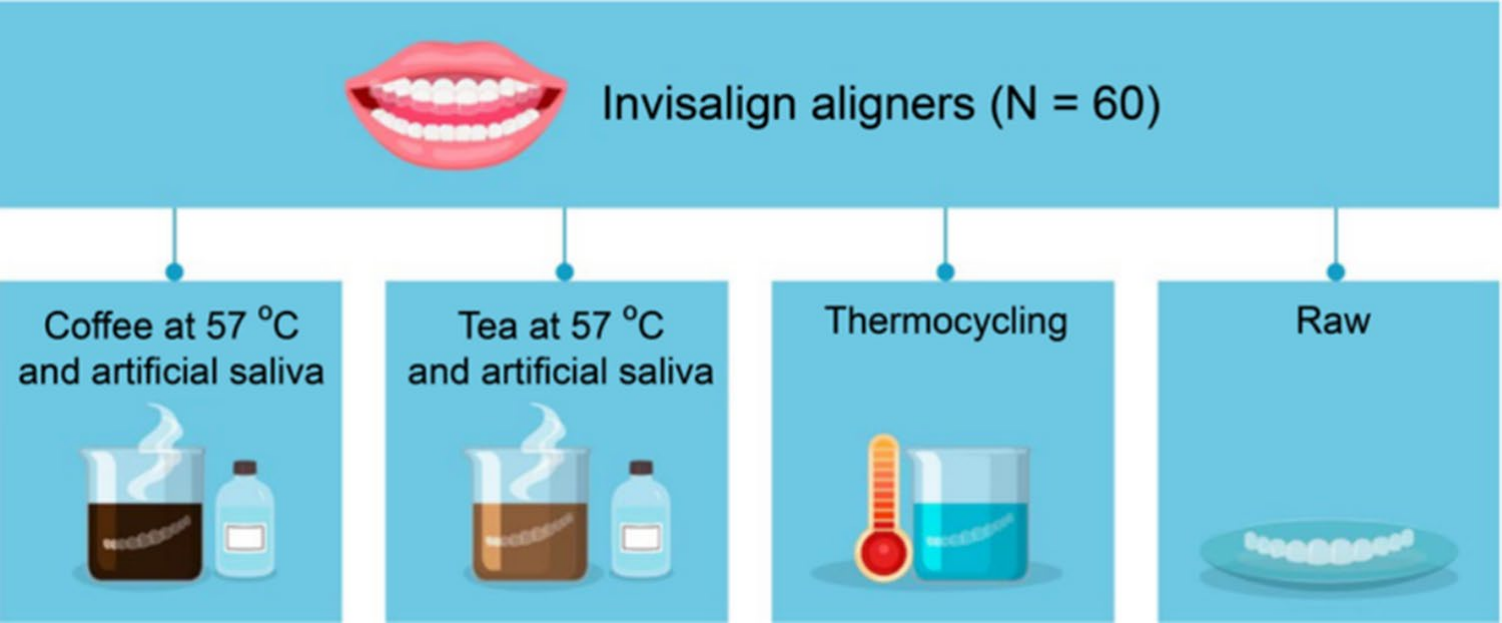
Summary of the specimens grouping based on different treatment conditions, including exposure to tea, coffee, thermocycling, and untreated (raw) aligners

After the thermocycling, the specimens were divided into four groups (Fig. 2). Specimens from the first group were immersed into a solution of 30 g of instant coffee powder (Nescafé® Original, Nestlé, Vaud, Switzerland) in 2.5 L of distilled water, and specimens from the second group were immersed in a solution of 9 bags of tea (English Breakfast Tea, Twinings, Andover, UK) in 2.5 L of distilled water (Bernard et al. 2020). Both groups were maintained at a thermostat-regulated temperature of 57 °C, which is the safe temperature for consuming hot beverages (Fig. 2). A 6DOF (6 degree of freedom) aluminum robotic arm equipped with a precision clip at its end-effector is designed for handling specimens with high accuracy. The arm is constructed from lightweight yet durable aluminum, The arm is powered by six high-precision motors, each controlling one degree of freedom, ensuring both strength and smooth operation.

The robotic arm is controlled by a controller with an accurate timing system, allowing it to execute movements and operations with consistent precision. The controller is pre-programmed to perform the experiment with strict timing and motion accuracy, ensuring repeatability and reliability throughout the process.

**Fig. 3:**
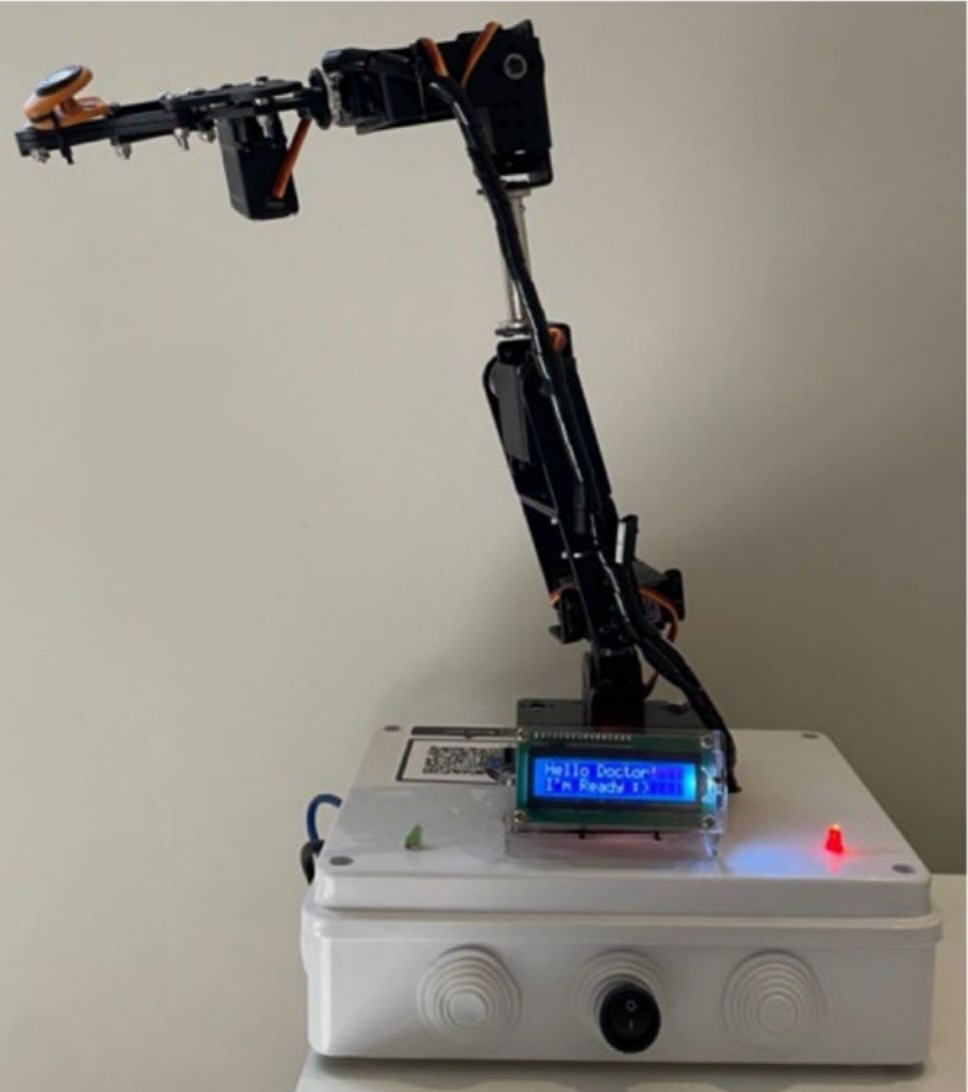
A specially designed robot to hold each specimen

This setup enables the robot to handle delicate specimens efficiently, making it ideal for automated experiments, laboratory applications, and industrial processes requiring precision handling. Each specimen was immersed once in each solution and then in 500 mL of artificial saliva to simulate typical intermittent drinking behavior. This cycle was repeated 200 times, with each immersion lasting 2 s (Fig. 3). The specimens were then rinsed under running water for 30 s and dried with a water-absorbent material prior to analysis and comparison with the raw and thermocycled groups. The third and fourth groups consisted of the raw and thermocycled specimens, respectively.

### Vickers indenter

**Fig. 4:**
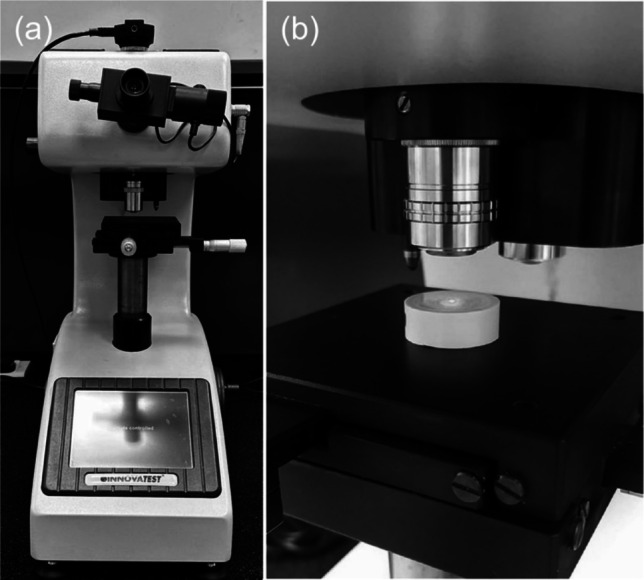
(**a**) Hardness testing with the Vickers indenter (INNOVATEST, FALCON 450, Deutschland GmbH); (**b**) specimen embedded in a plastic mold and prepared for testing

For the indentation process, each specimen was placed in a plastic mold with orthodontic plaster. Using a Vickers indenter (INNOVATEST, FALCON 450, Deutschland GmbH, München, Germany), the microhardness was measured by applying a 25-gf load on each specimen for 15 s (Fig. 4a and b). Three indentations at a distance of approximately 100 μm were made in the center of each specimen (Alhendi et al. 2022). Microscopy was used to measure the size of each indentation. Vickers hardness (VHN) was calculated using the following formula:

$$\text{VHN}=1.854\left(\text{F}/\text{D}2\right)$$

where D2 is the indentation area in square millimeters, and F is the applied load in kilogram force.

### Nanoindentation testing

**Fig. 5:**
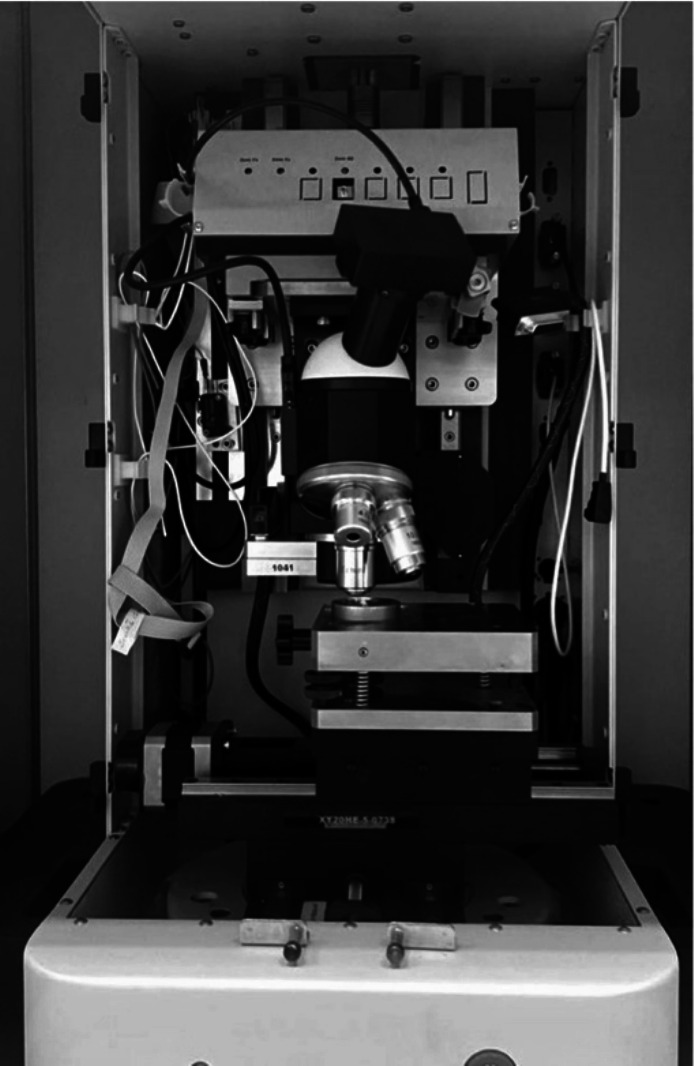
Nanoindentation testing using a Berkovich indenter (UMT1, Bruker, CA, USA)

All the nanoindentation tests on the surface of each specimen were performed using a Berkovich indenter (UMT1, Bruker, CA, USA) with a peak load of 10 mN (Fig. 5) (Iijima et al. 2015). Four indentations were made on each specimen by using a pyramidal diamond indenter. The elastic modulus was calculated in gigapascals (GPa) using proprietary software provided by the nanoindentation device.

## Data Analysis

Data were analyzed using the IBM SPSS Statistics software for Windows version 26.0 (IBM Corp., Armonk, NY, USA). Descriptive statistics (means and standard deviations) were used to describe the hardness and elastic modulus values. One-way analysis of variance was used to compare the mean values of hardness and elastic modulus among the four study groups. Subsequently, a post-hoc multiple comparison test (Tukey’s test) was used for pairwise comparisons of mean values. Statistical significance was determined at a *p*-value < 0.05.

## Results

A comparison of the mean values of hardness among the study groups (raw, thermocycling, tea, and coffee) showed statistically significant differences (F = 2576.38, *p* < 0.0001). Post-hoc multiple comparisons indicated that the mean hardness of the raw specimens was significantly higher than those of the other three groups (*p* < 0.0001 for each). In addition, the mean hardness of the thermocycling group was significantly higher than those of the tea and coffee groups (both *p* < 0.0001). The mean hardness of the tea group was significantly lower than those of the raw and thermocycled groups (both *p* < 0.0001), but not significantly different from that of the coffee group (*p* = 1.0) (Tables 2 and 3).

**Table 2:** Comparison of the mean values of hardness among the four study groups

Study Group	Mean	Standard Deviation	F-value	*p*-Value
Raw	5.5933	0.079	2576.38	< 0.0001
Thermocycling	4.2667	0.111		
Tea	3.7244	0.057		
Coffee	3.7244	0.080

**Table 3:** Multiple comparisons of mean values of hardness in pairs of the four study groups using a post-hoc test

Study Group	Study Groups	Mean Difference	*p*-Value	95% Confidence Interval	
				Lower Bound	Upper Bound
Raw	Thermocycling	1.32667	< 0.0001	1.2533	1.4000
	Tea	1.86889	< 0.0001	1.8090	1.9288
	Coffee	1.86889	< 0.0001	1.8090	1.9288
Thermocycling	Raw	− 1.32667	< 0.0001	− 1.4000	− 1.2533
	Tea	0.54222	< 0.0001	0.4823	0.6021
	Coffee	0.54222	< 0.0001	0.4823	0.6021
Tea	Raw	− 1.86889	< 0.0001	− 1.9288	− 1.8090
	Thermocycling	− 0.54222	< 0.0001	− 0.6021	− 0.4823
	Coffee	0.00000	1.00	− 0.0423	0.0423
Coffee	Raw	− 1.86889	< 0.0001	− 1.9288	− 1.8090
	Thermocycling	− 0.54222	< 0.0001	− 0.6021	− 0.4823
	Tea	0.00000	1.00	− 0.0423	0.0423

A comparison of the mean values of the elastic modulus among the four study groups (raw, thermocycling, coffee, and tea specimens) revealed statistically significant differences (F = 3.496, *p* = 0.025). The post-hoc multiple comparisons of the mean values indicated that the mean value of the elastic modulus of the raw group was significantly lower than that of the coffee group (*p* = 0.018) but not significantly different from those of the thermocycling and tea groups (*p* = 0.413 and *p* = 0.309, respectively). Furthermore, there were no significant differences in the mean elastic moduli of the pairs in the remaining groups (Tables 4 and 5).

**Table 4:** Comparison of mean values of elastic modulus among the four study groups

Study Group	Mean	Standard Deviation	F-Value	*p*-Value
Raw	0.8739	0.031	3.496	0.025
Thermocycling	1.1059	0.217		
Tea	1.0874	0.235		
Coffee	1.2518	0.270

**Table 5:** Multiple comparison of mean values of elastic modulus in pairs of the four study groups using a post-hoc test

Study Group	Study Groups	Mean Difference	*p*-Value	95% Confidence Interval	
				Lower Bound	Upper Bound
Raw	Thermocycling	− 0.232074	0.413	− 0.63249	0.16834
	Tea	− 0.213611	0.309	− 0.54055	0.11333
	Coffee	− 0.377981	0.018	− 0.70492	− 0.05103
Thermocycling	Raw	0.232074	0.413	− 0.16834	0.63249
	Tea	0.018463	0.999	− 0.30847	0.34540
	Coffee	− 0.145906	0.630	− 0.47284	0.18103
Tea	Raw	0.213611	0.309	− 0.11333	0.54055
	Thermocycling	− 0.018463	0.999	− 0.34540	0.30847
	Coffee	− 0.164369	0.240	− 0.39555	0.06681
Coffee	Raw	0.377981	0.018	0.05103	0.70492
	Thermocycling	0.145906	0.630	− 0.18103	0.47284
	Tea	0.164369	0.240	− 0.06681	0.39555

## Discussion

Concerns have been raised regarding the possibility that the mechanical properties of thermoplastic materials are affected by the temperature changes occurring in the oral cavity. In a previous study, thermocycling was performed for 1000 cycles, which is equivalent to 36.5 days of clinical service (Gale and Darvell 1999). Thermal changes do not affect the force delivery capabilities of thermoplastic overlay orthodontic appliances when used in clinical settings for two to three weeks (Kwon et al. 2008). Therefore, in this study, the thermocycling process was set to 500 cycles for approximately 14 days of clinical usage.

The mechanical characteristics of a material regarding its resistance to a particular load (indentation or penetration) are referred to as its hardness (Comba et al. 2020). Several factors, including the thickness of the material, thermoforming process, polymer structure, and polymerization, can affect the material quality (Sakaguch et al. 2019). In the present experiment, the Vickers indenter was used to assess the polymeric material’s hardness (Low and Shi 1998). The Vickers hardness values of the raw specimens in our study are consistent with those observed in a previous study that measured the hardness of Invisalign materials (Barone et al. 2017). In our study, the hardness test results showed that the hardness of Invisalign clear aligners decreased significantly after thermocycling and exposure to beverages at 57 °C. According to a previous report, an increase in oral temperature from 23 °C to 37 °C led to a decrease in hardness (Gould et al. 2009). Meanwhile, another study showed that the crystal structure of aligners is altered because of exposure to orthodontic forces and heat in the oral cavity, causing an increase in hardness and hyperplasticity after use (Condo et al. 2018).

However, a few previous studies have presented conflicting data regarding the relationship between age and hardness. In contrast to our findings, two studies concluded that aligner hardness increased after intraoral aging of the extracted sample (Schuster et al. 2004; Dalaie et al. 2021). Bradley et al. (2016) also reported a reduced hardness of Invisalign after a usage duration of 44 ± 15 days. Schuster et al. (2004) found that the hardness of the same aligners increased after 14 days of intraoral aging.

Our findings demonstrated that the raw Invisalign material had a significantly higher hardness value before the thermocycling aging process. However, after exposure to both tea and coffee at a temperature of 57 °C, the hardness values of the material significantly decreased. The observed decrease in hardness values of Invisalign aligners suggests a possible loss in their mechanical strength, therefore reducing the efficacy of tooth movement and increasing susceptibility to deformation or fracture. This result corresponds to earlier research showing a significant decrease in aligner hardness with intraoral aging (Mei et al. 2024).

Other investigations, however, have indicated a post-aging increase in hardness, presumably from changes in polymer crystalline structure or material cold working during mastication (Dalaie et al. 2024). These discrepancies may stem from differences in aging durations, environmental conditions, or measurement methodologies across studies.

Fang et al. (2020) reported an elastic modulus of 0.848 ± 0.063 GPa for Invisalign materials, which is consistent with our result of 0.8739 GPa. The aging process involving 500 thermocycling cycles with tea at 57 °C did not substantially change the elastic modulus, whereas this value was significantly lower than that obtained through a similar aging process with coffee.

A previous study examined the changes in the properties of the Invisalign LD30 material before and after two weeks of intraoral use. Although no substantial changes in the mechanical properties were observed, changes in the elastic modulus and an increase in stress relaxation were observed (Fang et al. 2020). These changes can be attributed to exposure to the oral environment, as well as changes in the crystallinity of the thermoplastic overlay orthodontic appliance under masticatory loads (Kwon et al. 2008).

Thus, the findings of this study indicate that elevated beverage temperatures significantly change the mechanical properties of Invisalign clear aligners. A recent in vitro study revealed the chemical composition and performance of clear aligners after exposure to various chemical liquids*.* However, continuous exposure to certain beverages, such as orange juice and chlorhexidine mouthwash, increased tensile strength, whereas exposure to carbonated beverages resulted in increased hardness (Asefi et al. 2024). In the present study, while the hardness decreased, the elastic modulus increased, particularly in the aligners exposed to coffee at high temperatures, indicating an increased rigidity. This may result from chemical interactions between the aligner material and coffee components during the immersion process.

Although the findings of this study are not conclusive, they provide insights into the behavior of clear thermoplastic aligners under increased temperature and aging. However, this study has several limitations. Because the clinical setting differs from the simulated oral conditions used in this investigation, the findings should be interpreted with caution. Thus, the in vitro study did not accurately simulate the typical 10-day aging process and exposure to high temperatures in an actual oral environment. Our aligners were not subjected to oral bacteria or enzymes, nor were they exposed to chewing, removal, reinsertion, or parafunctions such as bruxism. Further investigation of the potential states of various polymers after prolonged exposure to high temperatures may provide a deeper understanding of the observed outcomes.

## Conclusions

This study examined the impact of thermocycling and exposure to beverages at a high temperature of 57 °C on Invisalign clear aligners. A significant decrease in hardness was observed, suggesting that the resistance of the aligners to deformation was compromised under these conditions. Additionally, the coffee-exposed group exhibited an increased elastic modulus, indicating greater rigidity. Such changes in the material can affect the durability and effectiveness of orthodontic treatments. These findings highlighted the importance of considering environmental factors and dietary habits in the performance and longevity of Invisalign aligners and emphasize the need for further research to mitigate these effects.

## Data Availability

The data presented in this study are available upon request from the corresponding author.
